# Possible Role of Myopia as a Risk Factor for Mechanical Neck Pain in Medical Students : A Pilot Study

**DOI:** 10.31661/gmj.v8i0.1287

**Published:** 2019-01-01

**Authors:** Bahareh Kardeh, Alireza Ashraf, Sina Kardeh

**Affiliations:** ^1^Student Research Committee, Shiraz University of Medical Sciences, Shiraz, Iran; ^2^Shiraz Geriatric Research Center, Shiraz Burn Research Center, and Department of Physical Medicine and Rehabilitation, Shiraz University of Medical Sciences, Shiraz, Iran; ^3^Cellular and Molecular Medicine Student Research Group, Medical School, Shiraz University of Medical Sciences, Shiraz, Iran

**Keywords:** Myopia, Neck Pain, Pain Measurement, Refractive Errors, Visual Acuity

## Abstract

**Background:**

Mechanical neck pain (MNP) is a common health concern. Some studies suggested a relationship between neck pain and visual activity. We assessed the role of myopia as a contributing factor in the development of chronic MNP.

**Materials and Methods:**

All medical students at Shiraz University School of Medicine, who were 18 to 22 years old, were invited to participate in this retrospective case-control study from March 2016 to March 2017. Numeric Pain Rating Scale (NPRS) was used to assess the average neck pain severity over the past 6 months in order to enroll participants as the case (≥3) or control (<3) groups. Demographic data and medical profile were obtained. After identifying eligible cases, we assigned age- and sex-matched controls, who also met the study criteria. Subsequently, participants completed the Neck Disability Index (NDI) and Neck Pain and Disability Scale (NPDS) questionnaires and were evaluated for myopia severity. Variables were compared between the case and control groups as well as within the case group. P-value<0.05 was considered statistically significant.

**Results:**

From over 700 medical students, around 150 cooperated. Eventually, 31 eligible cases (and 31 controls) were studied. NDI and NPDS were significantly higher in the case group (P<0.001). However, no significant differences were noticed between the groups regarding the severity (P=0.123) and the duration (P=0.417) of myopia. Also, the correlation of myopia severity with NDPS (ρ=0.159, P=0.216) and NDI (ρ=0.201, P=0.116) was non-significant within the case group.

**Conclusion:**

Our findings have not supported the influential role of myopia in the development of chronic MNP.

## Introduction

It is estimated that over a third of billion people experience chronic neck pain annually [1]. Neck pain has constantly been among the top ten causes for disability-adjusted life-years (DALYs) over the past decades [2]. This condition substantially disrupts the patients’ well-being [3] and decreases their health-related quality of life [4]. Also, sickness leave and treatment costs add to the disease burden on a financial level [5]. A neck pain without apparent radiculopathy, myelopathy, or any definite underlying pathogenesis is known as the mechanical neck pain (MNP) [6]. A wide spectrum of individual, mechanical, and psychosocial predisposing factors, such as female gender, older age, prolonged sitting and poor postures, monotonous work tasks, depression, and occupational stress are associated with neck pain [7-11]. More recently, the correlation between ocular and musculoskeletal symptoms has become the focus of some studies. A large population study in 2011 on employees who worked with computers for at least an hour/day showed that ocular symptoms, combined exposure to spectacles and a visual acuity <1, and vergence disparity were risk factors for aggravation of neck/scapular area symptoms [12]. The suggested functional link between visual activity and neck muscles has encouraged researchers to shed light on the unclarified mechanisms that might be involved [13, 14]. Nonetheless, the evidence is still very limited and the actual impact of daily near-work activities in the long-term remains indeterminate. To this date, no study has examined chronic MNP in myopic general population by the exclusion of potential confounding factors. With this concern, we designed the present study to investigate the correlation between the severity of myopia and chronic MNP.

## Materials and Methods

### Participants and Settings

All medical students at the Shiraz University of Medical Sciences, Shiraz, Iran, who aged 18 to 22 years old, were invited to participate in this retrospective case-control study. Recruitment was conducted from March 2016 to March 2017. Exclusion criteria consisted of using glasses or contact lenses while engaging in near-work activities, onset of myopia and neck pain within the past 6 months, other ophthalmological diseases except for myopia, history of refractive corrective surgeries, history of neck or head trauma, history of neurological, musculoskeletal or collagen vascular diseases (such as chronic pain syndrome), malignancy, psychiatric disorders including depression, and history of receiving medical, manual or surgical therapeutic modalities other than occasional over the counter or traditional analgesics. Participants were asked to rate their average neck pain severity over the past 6 months using the Numeric Pain Rating Scale (NPRS). Based on this score, they were allocated to either the case (NPRS≥3) or the control (NPRS<3) groups.

### Data Gathering

Demographic data, characteristics of neck pain, and relative information on the past medical history, onset of myopia, duration of near-work activities per day as well as other potentially influential factors were obtained. With the aim of eliminating confounding factors as much as possible and ensuring a homogenous population, further requirements were set; these included utilizing a desk and chair for reading/studying and working with a computer, complying with standard postures, and negative history of vigorous physical activities. After identifying eligible subjects for the case group, we assigned age- and sex-matched controls from the initial participants, who also met the study criteria. Subsequently, all participants completed the validated Persian Neck Disability Index (NDI) and the Neck Pain and Disability Scale (NPDS) questionnaires [15]. In addition, they were evaluated for the severity of myopia by a single optometrist. Several self-report measures are used to assess pain. The NPRS-11 is a numeric scale with excellent reliability [16]. It has two defined ends; zero for no pain and 10 for the worst pain imaginable. NDI is a well-established 10-item questionnaire, which was originally created as an adaptation of the Oswestry Low Back Pain Disability Questionnaire [17, 18]. It inquires about symptoms (pain severity, headache, concentration, and sleeping), routine daily tasks (lifting, work, driving, and recreation), and optional activities (personal care and reading). Each question has 0-5 points; thus, making up a total of 0-50 [19]. NDI is often considered as the gold standard for other questionnaires [20]. NPDS has 20 items (200 points) that evaluate neck mobility, neck pain severity, the impact of neck pain on sentiment and cognition, and the degree of disruption in daily activities [21]. Higher scores indicate greater disability. Both questionnaires have strong a correlation and a good validity for chronic non-traumatic neck pain [22]. NDI and NPDS have been translated into Persian and validated by Mousavi *et al*. Authors considered these questionnaires to be superior due to their comprehensibility, internal consistency, easy administration, and suitability, as well as being time-saving for both patients and examiners [15].

### Ethical Considerations

Informed consent was obtained from the participants and the confidentiality of patients’ information was preserved. The study protocol was in accordance with the Helsinki Declaration and approved by the institutional review board.

### Statistical Analysis

Statistical analysis was performed using Windows SPSS software version 20 (SPSS, Inc., Chicago, IL). Data are represented descriptively as frequency and mean ± standard deviation (SD), and odds ratio (OR) as applicable. Mann–Whitney U test was used to compare variables between the case and control groups. Also, the correlation of myopia severity with NDI and NPDS within the case group was examined using Spearman’s rho. All tests were two-sided and a P-value<0.05 was considered statistically significant.

## Results

Over 700 1^st^ year to 4^th^-year medical students were invited to participate in this study. Approximately, 150 filled out the data sheets, of whom 47 had NPRS≥3. Eventually, 31 students fulfilled the criteria of study and were allocated to the case group. This group consisted of 23 (74.2%) female and 8 (25.8%) male students with the mean age of 19.93±1.59 years (Table-1). Although NPDS and NDI were significantly higher in the case group (P<0.001), the differences between myopia parameters were not significant (Table-2). Using logistic regression, we found that the outcome; i.e. neck pain, was not significantly influenced by either the severity (OR=1.65, 95%CI=0.87-3.11, P=0.122) or the duration (OR=0.913, 95%CI=0.68-1.21, P=0.534) of myopia. Using Spearman’s rho, the correlations of myopia severity with NDI (ρ=0.201, P=0.116) and NDPS (ρ=0.159, P=0.216) within the case group were found to be non-significant. Similarly, the duration of near-work activities did not show any significant impact on the NDI (ρ=0.199, P=0.121) and NDPS (ρ=0.213, P=0.096) scores.

## Discussion

The present study aimed to explore the possible role of myopia in the development of chronic MNP among a population of young adults. Although NDPS and NDI scores were significantly higher in the case group, the duration and severity of myopia were not significantly different. This finding is contrary to our hypothesis. Also, the average duration of near-work activities per day, which could be a potentially confounding factor, show no statistical difference between the case and control groups. Pain measurements are generally subjective and pain sensitivity has a fundamental correlation with gender [23, 24]. However, we had matched the groups in this regard. The disproportionate male to female ratio in our study can be attributed to the overall female-dominant classes at our medical school, the greater willingness of female students to cooperate, and a higher tendency among women to report pain as compared to men. Also, none of the participants had been diagnosed with psychiatric disorders or reported related symptoms of psychiatric problems such as depression. As the study population was homogeneous, it can be assumed that our participants were exposed to almost similar stressors. To the best of our knowledge, research in this area is scarce and mostly limited to experimental findings. In a study by Lodin *et al*, 33 healthy subjects completed a visually arduous task by viewing a monitor screen using four optical lenses at each time: binocular -3.5 D, monocular -3.5D, +3.5 D, and 0 D. Participants sat in an office chair that provided individually adjusted neck support. They reported their perceived eye- and neck/shoulder discomfort at the beginning as well as after each task. Neck/shoulder discomfort significantly elevated from baseline level throughout the experiment. Yet, the authors mentioned that the role of static posture cannot be ignored [25]. In a more in-depth study by Zetterberg *et al*, the association of eye and neck/shoulder discomfort was evaluated in 33 cases with chronic neck pain and 33 controls while performing four visual tasks using different trial lenses each time. While astigmatism, accommodation response, and internal eye discomfort (related to accommodative strain) contributed to the neck/shoulder discomfort, external eye discomfort (related to dry-eye) did not have any significant adverse impact [14]. In a similar study, Zetterberg *et al* examined the specific effect of eye-lens accommodation on trapezius muscle activity in 33 controls and 33 patients with neck pain, who also completed near-work viewing tasks using four distinct trial-lens. Trapezius muscle activity was not influenced by the lens accommodation alone. However, when there was incongruence between accommodation and convergence, a significant positive correlation was found between trapezius muscle activity and eye-lens accommodation, which subsequently leads to near-work-related neck discomfort [26]. Richter *et al* induced four levels of the oculomotor load in 28 subjects to study the trapezius muscle activity and reported that this parameter remained at a steady level in the absence of accommodative compensation. In contrast, a slope was observed when compensation occurred. Furthermore, the convergence response did not influence the electromyography of trapezius muscle [27]. Another study by Richter *et al*, which was conducted in a similar setting, investigated the impact of sustained oculomotor load on the static trapezius muscle activity in 28 subjects while staring at a viewing target (placed at a 5 diopter distance from the person’s near point of accommodation) through -3.5 and 0 diopter lenses for a duration of 5 minutes at each episode. The results showed that requiring a greater accommodative response induced a greater static trapezius muscle activity, which can consequently lead to trapezius muscle myalgia [28]. Frosman *et al* found a weak correlation between the timing in alternate near-far lens accommodation through four different lenses and the trapezius muscle activity. He attributed this correlation to a possible connection between the ciliary and trapezius muscles as well as the need for a more stable head posture during challenging visual tasks [29]. Valentino *et al* demonstrated that the movements of extra-ocular muscles in myopic patients result in a considerably different tonic activity of the trapezius and sternocleidomastoid muscles as compared to subjects with normal visual acuity. In myopic patients, the electromyography responses of neck muscles were not well-balanced [13]. Richter has discussed the impacts of high accommodation/vergence demands on neck pain. He suggested that oculomotor load and activation of the neck and scapular muscles have a close functional relationship; however, the mechanism(s) that relate these two systems need to be elucidated through the study of both clinical and basic aspects [30]. MNP is a multifactorial disorder. Therefore, an isolated investigation of a single factor can pose a methodological challenge. To avoid age-related structural changes that can cause neck pain, we studied a homogenous sample of young adults. However, severe neck pain or myopia is less common in such a population and a higher cutoff point for case inclusion would have yielded even less eligible cases [31]. Obviously, a larger sample size could increase the statistical power of this pilot study. Moreover, cases with severe myopia might often use their glasses or attain poor posture while doing near-work activities. Furthermore, the retrospective approach renders our study susceptible to recall bias. We suggest that additional objective assessments might improve the reliability of the results and offer new insights into this issue.

## Conclusion

The present study showed no correlation between impaired visual acuity and the development of chronic MNP in medical students. Nonetheless, the extent of consistent experimental findings on the impact of visual activities including accommodation, convergence, and extra-ocular muscles movements on the neck/shoulder discomfort cannot be ignored. In fact, these pieces of evidence seem to be sufficient to call upon further research for exploring their clinical significance.

## Acknowledgment

This article was extracted from the medical thesis written by Dr. Bahareh Kardeh, which was financially supported by the vice chancellor of research at Shiraz University of Medical Sciences, Shiraz, Iran (Grant No. 94-01-64-9905). The authors also thank the research consultation center of Shiraz University of Medical Sciences for proofreading this article.

## Conflict of Interest

Authors declare that they have no conflicts of interest.

**Table 1:** Demographic Data of the Participants

**Parameters**		**Total**
**Age (mean±SD, min-max)**		19.93±1.59 (18-22)
**Gender (n, %)**	Male Female	8 (25.8%) 23 (74.2%)
**Education Years (n, %)**	1^st^ 2^nd^ 3^rd^ 4^th^	9 (29%) 8 (25.8%) 7 (22.6%) 7 (22.6%)
**Past Medical History**		Non-significant
**Past Surgical History**		Non-significant
**Past Drug History**		Occasional analgesics

**Table 2:** Comparison of Variables between Case and Control Groups. All the Data Are Expressed as Mean±SD

**Parameters**	**Case group**	**Control group**	**Total**	P-value*
**NRPS score**	3.51±0.62	1.12±0.61	2.32±1.35	<0.001
**Near-work Duration, hour/day)**	7.38±2.76	6.25±2.58	6.82±2.71	0.97
**Severity of Myopia, Diopter**	-1.87±1.48	-1.27±1.17	-1.57±1.36	0.123
**Duration of Myopia, y**	3.22±3.12	2.48±2.73	2.85±2.93	0.417
**NDI, mean±SD**	9.51±2.11	2.80±1.47	6.16±3.83	<0.001
**NPDS Score**	51.61±18.98	18.80±10.74	35.2±22.52	<0.001

* Mann–Whitney U test
